# Falls following discharge after an in-hospital fall

**DOI:** 10.1186/1471-2318-9-53

**Published:** 2009-12-01

**Authors:** Rick D Davenport, Georgeta D Vaidean, Carol B Jones, A Michelle Chandler, Lori A Kessler, Lorraine C Mion, Ronald I Shorr

**Affiliations:** 1 HSR&D/RR&D Center of Excellence, James A. Haley VAMC, 8900 Grand Oak Circle, Tampa, FL, 33637, USA; 2 Department of Pharmacy and Health Outcomes, Touro College of Pharmacy, 230 West 125th Street, Suite 430, New York, NY, 10027, USA; 3 Methodist Healthcare Foundation, 1211 Union Avenue, Suite 450, Memphis, TN, 38104, USA; 4 School of Nursing, Vanderbilt University, 461 21st Avenue South, Nashville, TN, 37240, USA; 5 Geriatric Research, Education, and Clinical Center (GRECC), Malcom Randall VAMC (182), 1601 SW Archer Road, Gainesville, FL, 32608, USA; 6 Department of Aging and Geriatric Research, University of Florida, 1329 SW 16th Street, Gainesville, FL, 32611, USA

## Abstract

**Background:**

Falls are among the most common adverse events reported in hospitalized patients. While there is a growing body of literature on fall prevention in the hospital, the data examining the fall rate and risk factors for falls in the immediate post-hospitalization period has not been well described. The objectives of the present study were to determine the fall rate of in-hospital fallers at home and to explore the risk factors for falls during the immediate post-hospitalization period.

**Methods:**

We identified patients who sustained a fall on one of 16 medical/surgical nursing units during an inpatient admission to an urban community teaching hospital. After discharge, falls were ascertained using weekly telephone surveillance for 4 weeks post-discharge. Patients were followed until death, loss to follow up or end of study (four weeks). Time spent rehospitalized or institutionalized was censored in rate calculations.

**Results:**

Of 95 hospitalized patients who fell during recruitment, 65 (68%) met inclusion criteria and agreed to participate. These subjects contributed 1498 person-days to the study (mean duration of follow-up = 23 days). Seventy-five percent were African-American and 43% were women. Sixteen patients (25%) had multiple falls during hospitalization and 23 patients (35%) suffered a fall-related injury during hospitalization. Nineteen patients (29%) experienced 38 falls at their homes, yielding a fall rate of 25.4/1,000 person-days (95% CI: 17.3-33.4). Twenty-three patients (35%) were readmitted and 3(5%) died. One patient experienced a hip fracture. In exploratory univariate analysis, persons who were likely to fall at home were those who sustained multiple falls in the hospital (p = 0.008).

**Conclusion:**

Patients who fall during hospitalization, especially on more than one occasion, are at high risk for falling at home following hospital discharge. Interventions to reduce falls would be appropriate to test in this high-risk population.

## Background

Patient falls represent over one-third of incidents reported in hospitals [1,2], and they are the largest single category of reported hospital adverse events [1-3]. Patient falls are more frequently reported than medication errors, equipment related incidents, and documentation errors [1]. There are significant costs associated with patient falls, including patient care costs [4], liability [5], and increased length of stay [4]. With approximately 2% to 7% of acute-care hospitalized patients experiencing at least one fall during their stay [3,6,7], researchers and health care institutions have placed prioritization on the development and implementation of in-hospital fall prevention strategies and programs. As a result numerous fall risk assessment tools have been developed to identify patients at risk of falling in hospitals [8,9], as well as the implementation of a wide range of hospital-based fall prevention programs [7,10,11]. While there is a growing body of literature on fall prevention in the hospital, the data examining the fall rate and risk factors for falls in the immediate post-hospitalization period has not been well described [12-14].

While there is very little data examining the fall rate and risk factors for falls in the immediate post-hospitalization period of the older adult [12-14], there are almost no data regarding the fall rate and risk factors for falls in the immediate post-hospitalization period among hospitalized patients who fall - a potentially vulnerable population. Therefore, the aims of the present study were to determine the fall rate of in-hospital fallers at home and to explore the risk factors for falls during the immediate post-hospitalization period of patients who had fallen during their hospital stay.

## Methods

### Study population

Methodist Health University Hospital (MHUH) has an ongoing Fall Evaluation Service as part of a quality improvement project. MHUH is a 652-bed urban community hospital in Memphis, Tennessee. The hospital provides primary to tertiary care to a diverse adult patient population. As previously described [15], MHUH uses a Fall Evaluation Service, which provides 24-hours/day, 7-days/week coverage of 16 medical/surgical nursing units and it allows for a greater detection of falls during hospitalization, than by incident reports. The Fall Evaluation Service consists of trained healthcare professionals (fall evaluators), who assess patients sustaining a potential fall event using a standardized data collection tool.

The Fall Evaluation Service team maintains a log of all hospitalized patient falls, which was used to identify potential participants for the present study. A prospective cohort of subjects who sustained a fall during an inpatient admission to MHUH between February and June 2006 were recruited. Inclusion criteria were: English speaking subjects who had fallen during this hospitalization, had not been a nursing home resident prior to hospitalization or would not be discharged to a nursing home, had a life expectancy of greater than 3 months, were alive at the time of hospital discharge, had a home phone, and had a next of kin available as a backup contact person. Because this study was used as a pilot/feasibility study to develop a home-based intervention to prevent falls in this population, we only included patients who lived 30 miles or less from the hospital. We did not restrict our study to older patients because in our experience, many younger hospitalized patients also exhibit falls due to frailty and would potentially benefit from a home based fall prevention intervention. Informed consent was obtained in accordance with the guidelines from the Methodist Healthcare Institutional Review Board, which approved the study.

### Baseline data collection

Data were collected using the fall evaluation service and medical record review. Initial assessment consisted of recording clinical and demographic characteristics, pre-hospitalization falls history, number of in-hospital falls, and whether any injuries were sustained during the in-hospital fall. Additional data included whether an ambulatory assistive device (e.g. walker, cane, and wheelchair) was utilized at home prior to hospitalization and whether participants were seen by physical and/or occupational therapy during their hospital stay.

### Follow-up and outcomes ascertainment

The main outcome was the occurrence of a self- or caregiver-reported fall at home during the 4 weeks after hospital discharge, a high risk time for a fall post-hospitalization [12]. Participants were contacted weekly by telephone beginning the participant's arrival to their home--either after being discharged from the hospital or after a short stay in a rehabilitation center or skilled nursing facility. Weekly phone calls consisted of a semi-structured interview conducted by health professional staff, trained in falls assessment and telephone interviewing techniques. To minimize participant burden, the content of the calls was limited to whether the subject remained at home, the number of falls, and the utilization of rehabilitation services during the seven day interval. The primary source of information was the participant, followed by the caregiver, if necessary.

Additionally all participants were provided a flyer on 'Preventing falls and fractures' as part of usual care services from the Fall Evaluation Services Program. The flyer outlined fall prevention tips, such as: having their vision and hearing checked, wearing rubber-soled shoes, and keeping pathways in their home cleared.

### Statistical analysis

Falls during the follow-up were assessed in two ways: 1) proportion of patients who fell, and 2) rate of falls per patient-day of follow up. Patient-days of follow-up began on the day of the participant's arrival home and ended at the first date of either death, admission to a nursing home, loss to follow-up, refusal to participate or end of study. Days rehospitalized were excluded from person-time.

We evaluated the effect of exposure variables on the proportion of persons who sustained a fall during the follow up using Chi-Square or the Fisher's exact test where appropriate. We estimated the effect of exposure variables on the rate of falling using the test for incidence rate ratio assuming a Poisson distribution and used Poisson regression for exploratory multivariate analysis with correction for overdispersion. All statistics were performed using SAS, version 9.1 (SAS Institute, Inc., Cary, NC) and all p-values were two-sided.

## Results

### Study population

**Figure 1:**
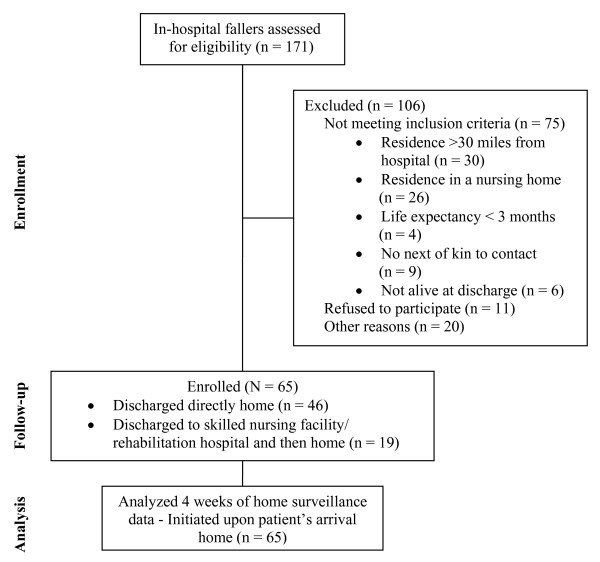
**Flow chart of enrollment, follow-up, and analysis**.

During the study period, 171 fallers were identified by the Fall Evaluation Service. Among these, 75 patients were not eligible for this study, 11 refused, and 20 not enrolled for other reasons (16 patients were discharged before screening, 2 patients the hospital staff asked not to approach due to risk management issues/hostile patient, 2 screens were missed) (Figure 1). Sixty-five patients agreed to participate and were enrolled in our study. Those who refused to participate (n = 11) were similar in age (mean 62 years, standard deviation ± 15), and race (64% African American), but were more likely to be male (82%).

The participating 65 patients had a mean age of 62.5 years (range 22-97), 43% were female, 75% were African American, and average hospital stay was 13.8 days. Of these, 46 (71%) were discharged immediately to home and 19 (29%) were discharge to a skilled nursing facility/rehabilitation hospital and then discharged home. Most participants (65%) had a history of falls prior to this hospital admission, with 32% reporting a history of multiple falls. All participants experienced a fall during their hospital stay (an inclusion criterion); additionally 16 (25%) of participants suffered multiple falls during their hospital stay, with 23 (35%) experiencing an injury secondary to the hospital fall (Table 1).

**Table 1:** Baseline characteristics of study participants

Characteristic	N (%)
Age, mean ± SD*, (range)	62.5 ± 13, (22-97)
Women	28 (43%)
Race/ethnicity	
African American	49 (75%)
Caucasian	16 (25%)
Number hospitalizations previous year	
0	27 (42%)
1	14 (22%)
2	10 (15%)
≥ 3	14 (22%)
History of falls prior to hospital admission	42 (65%)
Ambulatory assistive device utilized at home prior to hospital admission (e.g. walker, cane, and wheelchair)	30 (46%)
Diagnosis	
Hypertension	56 (86%)
Diabetes mellitus	34 (52%)
CHF	19 (29%)
Stroke	12 (19%)
Dementia	7 (11%)
Parkinson's Disease	1 (1.5%)
Multiple falls during current hospital stay	16 (25%)
Injury due to fall during current hospital stay	23 (35%)
PT/OT^† ^received during current hospital stay	44 (68%)
Duration of hospital stay	
< 7 days	15 (23%)
7-14 days	25 (38%)
≥ 15 days	25 (38%)
Discharged home	
Immediately	46 (71%)
After a short term skilled nursing facility/rehab hospital stay	19 (29%)

*SD = standard deviation.

†PT/OT = physical therapy/occupational therapy.

### Falls following discharge

Phone calls made each week to all the 65 participants. Information was obtained directly from the patient; in the circumstance when the participant was unavailable the next of kin was contacted regarding hospitalizations, nursing home placement, or mortality. All 65 participants initially consented to receive phone calls; only three (4.6%) participants refused phone contact at home after 1, 2 and 3 weeks respectively. These three participants never experienced a fall at home up to the point of their study discontinuation. Fifty three out of 58 respondents (91%) expressed a willingness to participate in future trials that involved a phone surveillance system to track their fall rates.

During the 4 weeks of surveillance, 19 (29%) participants suffered 38 falls at their homes. The 65 participants enrolled contributed 1498 person-days of follow-up (mean = 23 days/person), yielding a fall rate of 25.4 falls/1,000 person-days (95% CI: 17.3-33.4), for our full sample (age range 22-97). Fall rates were similar between age groups: 25.5 falls/1,000 person-days for our < 64 year-old subsample, and 25.2 falls/1,000 person-days for our > 65 year-old subsample. Sixty-three percent of the falls occurred during the initial 2 weeks after hospital discharge, with only 14 (37%) falls occurring during the final two weeks after discharge. One participant suffered a hip fracture. Twenty-three (35%) participants were readmitted to the hospital. There were 3 deaths (5%), and 4 (6%) nursing home placements. Fifteen participants (23%) received physical and/or occupational therapy at home during the 4 weeks of follow up.

### Comparison of fallers and non-fallers

Data examining the risk factors for falls in the immediate post-hospitalization period revealed no significant differences between the group of fallers and non-fallers in nearly all categories, including: age, use of ambulatory assistive device, previous hospitalizations, previous fall history, injury due to fall during hospital stay, and the duration of the index hospitalization. Additionally there were no significant differences found if participants were discharged to a skilled care facility prior to arriving home or if they received therapy (i.e., physical/occupational) services during or after their hospitalization. Males were not more likely to fall (p = 0.1), but sustained more falls once discharged (p = 0.033). Persons who suffered multiple falls during their current hospitalization were more likely to fall (p = 0.056) and sustained a much higher rate of falls once discharged (p = .008) (Table 2). After controlling for gender, the rate of falls following discharge remained higher among persons who fell more than once during hospitalization (p = 0.001).

**Table 2:** Characteristics of participants who suffered a fall compared to those who did not - Four weeks post-hospital discharge

Variable	Non-Fallers (n = 46) N (%)	Subjects who fell (n = 19) N (%)	*P*-value (fall)*	Person Days	Number of falls	*P*-value (rate)†
Age			0.584			0.981
< 65	24 (52%)	12 (63%)		824	21	
>= 65	22 (48%)	7 (37%)		674	17	
Gender			0.102			0.033
Male	23 (50%)	14 (74%)		846	30	
Female	23 (50%)	5 (26%)		652	8	
Multiple hospitalizations			0.397			0.766
Yes	19 (41%)	5 (26%)		518	12	
No	27 (59%)	14 (74%)		980	26	
History of falls prior to admission			0.401			0.148
Yes	28 (61%)	14 (74%)		1005	31	
No	18 (39%)	5 (26%)		493	7	
Assistive ambulatory devices at home			0.589			0.087
Yes	20 (43%)	10 (53%)		676	24	
No	26 (57%)	9 (47%)		822	14	
Multiple falls during hospital stay			0.056			0.008
Yes	8 (17%)	8 (42%)		381	19	
No	38 (83%)	11 (58%)		1117	19	
Injury due to fall during hospital stay			0.159			0.184
Yes	19 (41%)	4 (21%)		473	7	
No	27 (59%)	15 (79%)		1025	31	
PT/OT‡ during hospital stay			0.572			0.352
Yes	30 (65%)	14 (74%)		1047	30	
No	16 (35%)	5 (26%)		451	8	
Length of stay			0.417			0.107
< 14 days	26 (57%)	13 (68%)		934	30	
>= 14 days	20 (43%)	6 (32%)		564	8	
Discharge directly to home			0.389			0.72
Yes	34 (74%)	12 (63%)		1038	25	
No	12 (26%)	7 (37%)		460	13	
PT/OT after hospital stay §			0.751			0.208
Yes	10 (23%)	5 (26%)		413	6	
No	34 (77%)	14 (74%)		1064	32	

*Chi-square or fisher's exact test.

†Poisson regression.

‡PT/OT = physical therapy/occupational therapy.

§Missing data with this characteristic: n = 63 subjects with 1477 days of follow up.

## Discussion

The immediate post-hospitalization period represents a particularly high-risk time for adverse events [16,17], including a high risk for falls [12,13]. One study reported older adults who are discharged home after a medical illness have an increased fall rate in the first 2 weeks (8.0 falls per 1000 person-days) to 1 month (6.7 falls per 1000 person-days) [12], which is approximately two-fold higher than rates (range 3.7 to 4.2 per 1,000 person-days) seen for older adults in a hospitalized setting [3,6,18]. An additional study revealed that older adults who were discharged home with skilled-care (i.e., nursing or physical therapy) had a higher incidence (20% vs. 8%) of falls (compared with those who were not receiving skilled-care) within the first 30-days after hospitalization [13]. These two studies also prospectively defined the pre and post hospital discharge risk factors for falling in the immediate post-hospitalization period [12,13]. Pre-hospital discharge risk factors included: use of a walker, decline in mobility, dependency in activities of daily living (ADL), multiple falls during hospital stay, cognitive impairment, multiple hospitalization in the year prior, and post hospitalization risk factors include: self report of confusion, use of antidepressants, delirium, and poorer balance [12,13].

To our knowledge, this is the first study to specifically follow hospital fallers at home. Previous studies [12,13] have followed hospitalized patients home after discharge, however they did not identify whether the participant had a fall in the hospital prior to their discharge home. Our findings may have important clinical implications, as they indicate that during the immediate 2-4 week post-hospitalization period there is a high risk of falls for patients who sustained a fall during their hospital stay. Patients who sustain a fall during their hospital stay represent a considerable patient population as an estimated 2 to 7% of all acute care hospital admissions are reported to suffer a fall during their hospitalization [3,6,7].

Our findings are consistent with those of Mahoney who examined falls in the 65 year and older population [12], in that the number of falls were almost two-fold higher in the first two weeks after hospital discharge then in later weeks. However even though our sample population included middle-aged and younger participants, our rate of falls was considerably higher. Mahoney, for example reported 6.7 falls per 1000 person-days in the first 4 weeks after hospitalization while our fall rate was 25.4 falls per 1000 person-days for the full sample (age range 22-97) and 25.2 falls per 1000 person-days for our 65 year and older subsample after the first 4 weeks. This nearly four-fold higher fall rate may be attributed to our differing participant inclusion criteria in which we only enrolled patients who had fallen during their hospital stay. A higher fall rate in this subpopulation of hospital fallers is not unexpected given the numerous studies that have shown a history of falling during the previous year is a strong predictor of future falls [19-21].

The fall rate method is the most reliable method recommended for measuring the incidence of falls [22], however the fall rate statistic may artificially be inflated if the sample includes a small amount of participants who repeatedly fall. However when compared to previous studies our study had a considerably higher percentage of overall participants who fell. For instance, previous studies have revealed incidence of falling in the first 4 weeks after returning home as high as 15% in the 65 year and older population [12] and 14% in the 70 year old and older population [13]. While our incidence of falls was 29% for the full sample (age range 22-97) and 24% for our > 65-year-old sub sample after the first 4 weeks. Again this nearly two-fold higher percentage of overall participants who fell may be attributed to our differing participant inclusion criteria.

In our study, participants who had injured themselves while falling in the hospital were no more likely to fall at home. A possible interpretation of these results may be that participants who had recently injured themselves from a fall in the hospital may have become more cautious of falling again. Previous studies have shown that older adults who suffer an injury due to a fall are more fearful of falling [23,24] which can lead to more cautious protective behaviors that could contribute to the prevention of further falls [24].

Surprisingly, there was no difference in rate or proportion of fallers among older and younger participants. It is plausible that factors related to recovery from illness, rather than baseline measures of frailty, determine fall risk after hospitalization. Thus, interventions aimed at reducing falls after hospitalization should not necessarily focus on a specific age group.

Interestingly, those who went to a skilled nursing facility or rehabilitation center prior to going home and those who received physical and/or occupational therapy during or after hospitalization were not less likely to fall. However the participant's functional mobility level was not fully assessed at hospital admission/discharge, at skilled nursing facility/rehabilitation center, or upon arrival home. Prior research suggests that 'new' and 'recurrent' fallers are likely to have functionally deteriorated more during their hospital stay (i.e., bathing, dressing, toileting, bed-chair transfers) and have a higher risk of falling upon arriving home [13]. Additional research may help differentiate the level of functional recovery needed to prevent falls after hospital discharge.

Although this was a prospective study, it has several limitations. First, although our response rate (86%) and withdrawal rate (4.6%) in our study was very good, the generalizability of our study is limited as only 65 patients (38%) out of 171 in-hospital fallers were followed. Extrapolation to other settings may also be limited due to our small sample size (65) and our study taking place in a single urban tertiary care medical center. Also, the assessment of pre-hospitalization risk factors was based on self-report and potentially subject to recall bias. However, in a previous fall study [12], a comparison between participants' and proxies' recall of participants' pre-hospitalization risk factors found no obvious reporting bias. Third, the numbers of falls reported upon arrival home are self reported and the circumstances are not well known. It is possible that falls at home that do not result in injury are less likely to be reported than falls that do result in injury.

## Conclusion

As hospital length of stay has decreased, and transitions of health care have become increasingly fragmented, the post-hospitalization period represents a high-risk time. Previous studies have found that older adults who are discharged home after a medical illness have an increased fall rate [12,13]. Our results suggest that a high risk for falls is not limited to the 65 and older post-hospitalized population. All adults who have fallen during their hospitalization have a high rate of falls during their immediate post-hospitalization period, especially patients who have fallen on more than one occasion during their hospitalization. Our study suggests that weekly phone calls are well accepted by patients and may potentially be a feasible hospital-based surveillance system to monitor post-discharge fall risk factors and could be utilized to test future interventions that reduce falls in this high-risk population.

## Competing interests

The authors declare that they have no competing interests.

## Authors' contributions

RD contributed to the statistical analysis and interpretation of the data, and drafted and revised the manuscript. GV made substantial contributions to the conception and design of the study, acquisition of the original data and interpretation of the data. CJ lead in the acquisition of the original data. MC made substantial contributions to the conception and design of the study, acquisition of the original data. LK made substantial contributions to the conception and design of the study, acquisition of the original data and interpretation of the data. LM made substantial contributions to the conception and design of the study, and interpretation of the data. RS conceived and designed the study, performed the statistical analyses, drafted and revised the manuscript. RD, GV, LM, CJ, MC, LK, and RS all critically revised the manuscript. All authors read and approved the final manuscript.

## Pre-publication history

The pre-publication history for this paper can be accessed here:

http://www.biomedcentral.com/1471-2318/9/53/prepub
